# Effects of a Dielectric Barrier Discharge (DBD) on Characteristics of Polyaniline Nanoparticles Synthesized by a Solution Plasma Process with an Ar Gas Bubble Channel

**DOI:** 10.3390/polym12091939

**Published:** 2020-08-27

**Authors:** Jun-Goo Shin, Bhum Jae Shin, Eun Young Jung, Choon-Sang Park, Jae Young Kim, Heung-Sik Tae

**Affiliations:** 1 School of Electronics Engineering, College of IT Engineering, Kyungpook National University, Daegu 41566, Korea; bmw345@ee.knu.ac.kr (J.-G.S.); eyjung@knu.ac.kr (E.Y.J.); 2 Department of Electronics Engineering, Sejong University, Seoul 05006, Korea; hahusbj@sejong.ac.kr; 3 Department of Electronics and Computer Engineering, College of Engineering, Kansas State University, Manhattan, NY 66506, USA; purplepcs@ksu.edu; 4 Department of New Biology, Daegu Gyeongbuk Institute of Science & Technology, Daegu 42988, Korea; jyk@dgist.ac.kr

**Keywords:** solution plasma, polyaniline nanoparticle, dielectric barrier discharge, polymerization, gas bubble channel

## Abstract

The quality of polyaniline nanoparticles (PANI NPs) synthesized in plasma polymerization depends on the discharge characteristics of a solution plasma process (SPP). In this paper, the low temperature dielectric barrier discharge (DBD) is introduced to minimize the destruction of aniline molecules induced by the direct current (DC) spark discharge. By adopting the new electrode structure coupled with a gas channel, a low temperature DBD is successfully implemented in a SPP, for the first time, thus inducing an effective interaction between the Ar plasma and aniline monomer. We examine the effects of a low temperature DBD on characteristics of polyaniline nanoparticles synthesized by a SPP with an Ar gas bubble channel. As a result, both carbonization of aniline monomer and erosion of the electrode are significantly reduced, which is confirmed by analyses of the synthesized PANI NPs.

## 1. Introduction

Over the past decades, there has been considerable interest in the synthesis of nanomaterials due to unique electrical, optical, magnetic, and catalytic properties. Among the various methods for nanomaterial synthesis, the solution plasma process (SPP) is a simple and eco-friendly process because a plasma provides reactive chemical species and radicals without any strong chemical reagents [1,2,3]. However, it is very difficult to generate a discharge in liquid because its density is 10^4^ times higher than gas. In general, in a SPP, a discharge is formed in a liquid by applying a high voltage between a pair of pin-type metal electrodes with a narrow inter-electrode distance. Therefore, the strong direct current (DC) spark discharge is generated locally in the metal electrode region, whereby the electrode material is evaporated or sputtered, rapidly cooling in the liquid to form nanoparticles. Accordingly, the SPP has been mainly used to synthesize metal nanoparticles [4,5,6,7,8,9].

Recently, various applications using organic nanoparticles have been intensively studied [10,11,12,13,14,15,16,17]. In particular, polyaniline (PANI) has attracted a significant attention because it is applicable to various promising electronic devices such as supercapacitors, sensors, corrosion protective layers, and flexible displays due to such good features as good environmental and high chemical stability, thermal stability, low cost, and easy synthesis [18,19,20,21,22,23,24,25,26,27,28]. In general, since a strong DC spark discharge is formed in the conventional SPP, it is difficult to synthesize organic nanoparticles requiring low temperature discharge. In a previous study, we introduced the gas channel into the solution to synthesize organic nanoparticles by the SPP method. The pulsed DC streamer discharge within the Ar bubble channel could be stably generated with a significantly reduced firing voltage. It has also been demonstrated that polyaniline nanoparticles (PANI NPs) can be synthesized by SPP [29]. Nevertheless, a strong DC streamer discharge occurred between the two pin-type metal electrodes in the Ar bubble channel, resulting in contamination of the electrode erosion. In particular, the synthesized PANI NPs were observed to have a high carbon content. It is inferred that the Ar plasma energy is high enough to destroy aniline molecules. Therefore, in order to suppress destruction of aniline molecules, low-temperature discharge is essential. The dielectric barrier discharge (DBD) is a low-temperature discharge because the discharge current is limited due to the dielectric barrier, which is suitable to suppress carbonization of aniline monomers and expected to facilitate better properties of PANI NPs synthesized by SPP.

In this paper, we introduced a new electrode structure coupled with a gas channel for DBD and synthesized PANI NPs using DBD for the first time in SPP. The new electrode structure consists of the pin-type electrode in the quartz tube and another cylindrical electrode at the outside quartz tube (hereinafter, ‘DBD electrode structure’ or ‘DBD structure’). The characteristics of DBD in solution are monitored by using a high-speed camera, photo sensor amplifier, and intensified charge coupled device (ICCD). In particular, the radiative species present in the plasma as a result of interactions between the aniline monomer and Ar plasma are monitored by optical emission spectroscopy (OES). To characterize the PANI NPs, Fourier transform infrared (FTIR), field emission scanning electron microscopy (FE-SEM), high resolution transmission electron microscopy (HR-TEM), and X-ray diffraction (XRD) are also examined.

## 2. Materials and Methods

### 2.1. Experimental Setup

The plasma apparatus was made of a glass cylinder with an outer diameter (O.D.), inner diameter (I.D.), and height of 40, 34, and 80 mm, respectively. The tungsten electrode was located in the gas bubble channel inside the quartz tube where its diameter was 0.5 mm and its exact position extruded 1 mm from the end of a capillary quartz tube. On the other hand, the copper electrode was wrapped on the surface outside the quartz where its width was 5 mm. The copper electrode was equipped 3 mm away from the end of the capillary quartz tube. The gap between the two capillary quartz tubes was 2 mm. The Ar gas with high purity (99.999%, Linde Korea, Seoul, Korea) was used as a main gas for producing the plasma and supplied with a flow rate of 100 standard cubic centimeters per minute (sccm) controlled by a mass flow meter (MKS type 1179, range 2000 sccm, MKS Instrument Inc., Andover, MA, USA) and was fed through both capillary quartz tubes. The amount of liquid aniline monomer was 23 mL. A bipolar pulse with an amplitude of 8 kV_p-p_ and a frequency of 5 kHz was generated by a high voltage amplifier (20/20C-HS, Trek, Inc., Lockport, NY, USA) and a pulse generator (AFG-3102, Tektronix Inc., Beaverton, OR, USA). The bipolar pulse duty ratio was 100 μs and the process time for polymerization was 2 h.

### 2.2. Synthesis of Polyaniline

The PANI NPs were synthesized by the SPP with low temperature DBD. The synthesized PANI NPs were mixed with ethanol and purified using the centrifugal separator for 20 min at 10,000 rpm. Next, the PANI NPs were added to distilled water and rinsed using the centrifugal separator at the same condition. This cleaning process was repeated twice. Finally, the solid PANI NPs powder were obtained after being dried at 60 °C in oven for 12 h. In other words, the solid particles obtained from the plasma processed liquid were used as measurement samples for various analyses.

### 2.3. Voltage-Current (V-I) and Electrical Power Measurement

To examine the dielectric barrier discharge in the liquid state, the discharge voltage was measured by a high voltage probe (P6015A, Tektronix Inc., Beaverton, OR, USA) connected to the powered electrode, i.e., a tungsten electrode, and the corresponding discharge current was measured by a current probe (Model 4100, Pearson Elec. Inc., Palo Alto, CA, USA) connected to the grounded electrode i.e., copper electrode. Electrical power consumption was measured with a digital power meter (WT210, Yokogawa Electric, Tokyo, Japan) for different discharge structures, such as DBD and DC structure. The digital power meter was connected to a power source for measuring the electrical power supplied from the power source to the discharge area.

### 2.4. Optical Emission Spectroscopy

The radiative species present in the plasma as result of interaction between the aniline monomer and Ar plasma were monitored by an optical emission spectrometer (OES) (Ocean Optics USB 4000, response range: 200–1100 nm, optical resolution: ~1.5 nm, Ocean Optics Inc., Dunedin, FL, USA) during SPP with low temperature DBD. The OES system consisted of lens (Collimating Lenses, Ocean Optics Inc., Dunedin, FL, USA), optical fiber (P600-20 UV-VIS, range 300 nm–1.1 µm, Ocean Optics Inc., Dunedin, FL, USA) and OES. The light focused by the lens from the plasma region is transmitted into the OES through an optical fiber.

### 2.5. High Speed Camera

The evolution of the Ar gas bubble channel was monitored with a high speed camera (Phantom Miro C110, AMETEK, Wayne, NJ, USA). In this case, two lenses (Nikon AF Nikkor 105 mm, 1:2.8 D, and Nikon-AF 36 mm DG, Nikon, Tokyo, Japan) were used at 5000 frames per second (fps) with a shutter time of 200 μs. The resolution was 272 × 256.

### 2.6. Intensified Charge-Coupled Device

The low temperature DBDs during SPP for plasma polymerization were monitored by an intensified charge-coupled device (ICCD) camera (Princeton Instruments, PI-MAX 2) in both shutter modes with exposure times of 5 and 100 ms, respectively.

### 2.7. Scanning Electron Microscopy

The shapes of the synthesized PANI NPs were monitored by field emission-scanning electron microscopy (FE-SEM, SU8220, Hitachi Korea Co. Ltd., Seoul, Korea) with an accelerating voltage and current of 5 kV and 10 mA, respectively.

### 2.8. Transmission Electron Microscopy

High resolution images and selected area electron diffraction (SAED) of synthesized PANI NPs were measured by transmission electron microscopy (TEM). This measurement was conducted with a Titan G2 ChemiSTEM CS Probe (FEI Company, Hillsboro, OR, USA). Samples were dispersed in ethanol and obtained with a carbon-coated copper grid. Energy dispersive X-ray spectroscopy (EDS) (SU8220, Hitachi Korea Co. Ltd., Seoul, Korea) was performed to find elements of synthesized PANI NPs.

### 2.9. Fourier Transform Infrared Spectroscopy

The crystalline phase of the synthesized PANI NPs were measured by Fourier transform infrared spectroscopy (FTIR PerkinElmer, Waltham, MA, USA). The FTIR spectra were measured by averaging 128 scans at a wavenumber resolution of 0.6 cm^−1^ in the range from 600 cm^−1^ to 4000 cm^−1^ using the attenuated total reflection (ATR) mode.

### 2.10. X-Ray Diffraction

In order to analyze the crystalline structure of the synthesized PANI NPs powder, and impurities such as tungsten, the synthesized PANI NPs powder was evaluated by X-Ray diffractometer (XRD, D8 Discover Bruker, USA) at the Korea Basic Science Institute (KBSI, Daegu, Korea). In the XRD analysis, the data were acquired with 2θ angle ranging from 10° to 80° at 0.08° intervals and Cu-kα (λ = 1.54 Å) was used as the X-ray source.

## 3. Results

**Figure 1:**
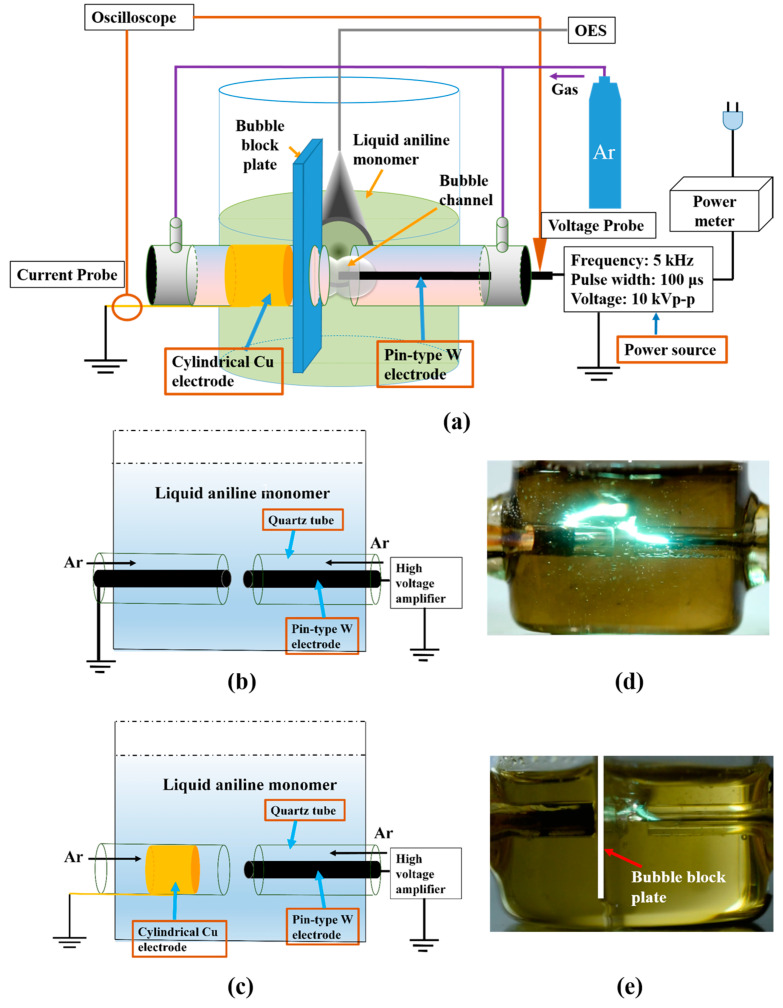
Experimental setup of the proposed DBD electrode structure with a grounded cylindrical copper electrode and a bubble block plate for plasma polymerization in liquid aniline monomer: (**a**) experimental setup, (**b**) DC electrode structure, (**c**) DBD electrode structure, (**d**) discharge image under DBD electrode structure without a bubble block plate, and (**e**) discharge image under DBD electrode structure with a bubble block plate.

Figure 1a shows the schematic diagram of an experimental apparatus with the proposed DBD electrode structure employed in this study. Since the discharge is very difficult to produce in a solution, especially a liquid aniline monomer with a high dielectric strength, the concept of a bubble channel has been proposed to be able to form a discharge in a gaseous state in a liquid aniline monomer [29]. Figure 1b shows a schematic of the DC electrode structure where the pair of pin-type tungsten (W) electrodes face each other in the previous study [29]. When the Ar gas was fed through both quartz tubes, an Ar bubble channel was formed to surround both of the W electrodes, thereby playing a significant role in providing a gaseous discharge path in liquid aniline. Accordingly, it was observed that a stable pulsed DC streamer occurred along the bubble channel at low applied voltage, which was not a conventional spark discharge in liquid. For effective polymer synthesis, both of the W electrodes were protruded into a liquid aniline to activate a reaction. As a result, the synthesis of PANI NPs using the SPP with the bubble channel was demonstrated. This DC streamer was still strong enough to induce the electrode erosion, including a carbonization. Figure 1c shows the proposed DBD electrode structure with the cylindrical copper (Cu) electrode outside the left quartz tube instead of the pin-type W electrode. Accordingly, the quartz tube (ɛ_r_ = 3.78) acted as a dielectric layer to capacitively limit a discharge current with an application of a bipolar pulse. In particular, the Cu electrode was 3 mm away from the end of quartz tube, thus preventing direct connection of the bubble channel between the W and Cu electrodes. Nonetheless, a strong DC streamer discharge could be produced in the outside region of the cylindrical Cu electrode due to charged gas bubbles during the plasma discharge, even under the DBD electrode structure, as shown in Figure 1d. For this reason, the bubble block plate was introduced into the left quartz tube, such that the stable DBD could be obtained thanks to the bubble block plate, as shown in Figure 1e. The bubble block plate was made of Teflon, and its dimensions were a width of 1 mm, a height of 113 mm and a length of 27 mm.

**Figure 2:**
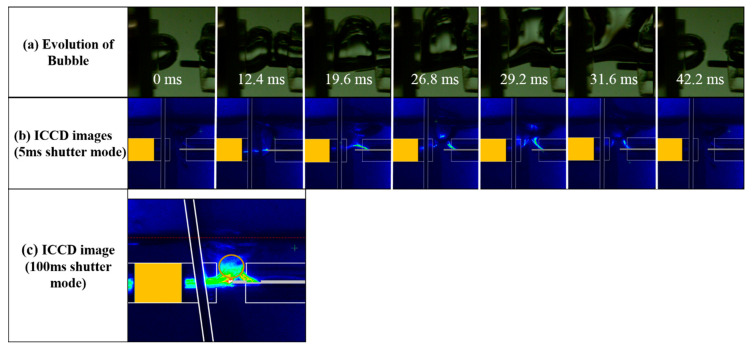
(**a**) Temporal evolution of the gas bubble in liquid aniline without discharge using a high-speed camera, (**b**) DBD images taken during exposure time of 5 ms in the shutter mode of an ICCD, and (**c**) a DBD image taken during exposure time of 100 ms in the shutter mode of an ICCD.

Figure 2a shows the temporal evolution of a gas bubble in liquid aniline without discharge, using a high-speed camera. The Ar gas injected from both tubes aggregated around the inlet in a bubble form to combine into a larger bubble, finally forming the gas bubble channel, which played a role in providing the discharge path in liquid aniline monomer between the cylindrical Cu and pin-type W electrodes. Then, immediately after forming the bubble channel, the Ar gas bubble was raised upwards due to the buoyancy, as shown in Figure 2a. The discharge was produced repeatedly only while the Ar gas bubble was moved upwards after forming a bubble channel, i.e., during the time period of about 19.2 ms ranging from 12.4 to 31.6 ms. No discharge was observed only under the liquid aniline condition, i.e., when the gas bubble channel was not formed. Accordingly, the discharge could be produced only during a period of gas-bubble channel formation, as shown in Figure 2a. We reported the temporal evolution of discharge formation within the bubble channel by a high-speed camera in the previous DC electrode structure [29]. In this study, however, the intensity of the DBD was too weak to take a discharge image using a high-speed camera, such that it was monitored with an ICCD camera.

Figure 2b shows the discharge images taken in a shutter mode with an exposure time of 5 ms using an ICCD camera. In the previous DC electrode structure, since the electric field was concentrated at the end of the W electrode, the discharge was mainly generated from the end of the cathode to the upper surface area of the anode along the Ar bubble channel in the discharge region placed between two quartz tubes [29]. In the proposed DBD electrode structure, however, the discharge path of a DBD must be created through the quartz tube under the cylindrical Cu electrode. Thus, unlike the DC structure in which the electric field was concentrated at the cathode, it was widely distributed inside the quartz tube surrounded by the cylindrical Cu electrode. As shown in Figure 2b, the discharge path was observed to ignite from the W electrode in the direction of the center axis of the quartz tube under the cylindrical Cu electrode. The DBD was observed only when the gas bubble channel was formed, as shown in Figure 2b.

Figure 2c shows the discharge image taken in a shutter mode with an exposure time of 100 ms, which corresponds to being around two bubble formation cycles. In Figure 2c, the most intense discharge was produced in the vicinity of the pin-type W electrode, whereas the discharge path was located in the direction of the center axis of the tube in the cylindrical Cu electrode. Furthermore, the discharge region was observed to be ascended due to the buoyance of the bubble channel. In particular, the active interaction between the Ar channel plasma and liquid aniline monomer for plasma polymerization would mainly occur in the ascended discharge region located in the region of the circle, shown in Figure 2c, where the OES was monitored. In DC discharge, the discharge current was maintained continuously during the application of the voltage [29]. On the contrary, the discharge current in the DBD flowed through the quartz, which essentially acted as a capacitor, charging with the opposite polarity. Therefore, DBD was spontaneously terminated due to the formation of the opposite charges accumulating in the quartz tube. In general, the analysis of instantaneous waveforms was very difficult because the bubble evolution and discharge formation were irregular in liquid aniline. Therefore, the current waveforms were averaged 100 times to analyze the overall trend.

**Figure 3:**
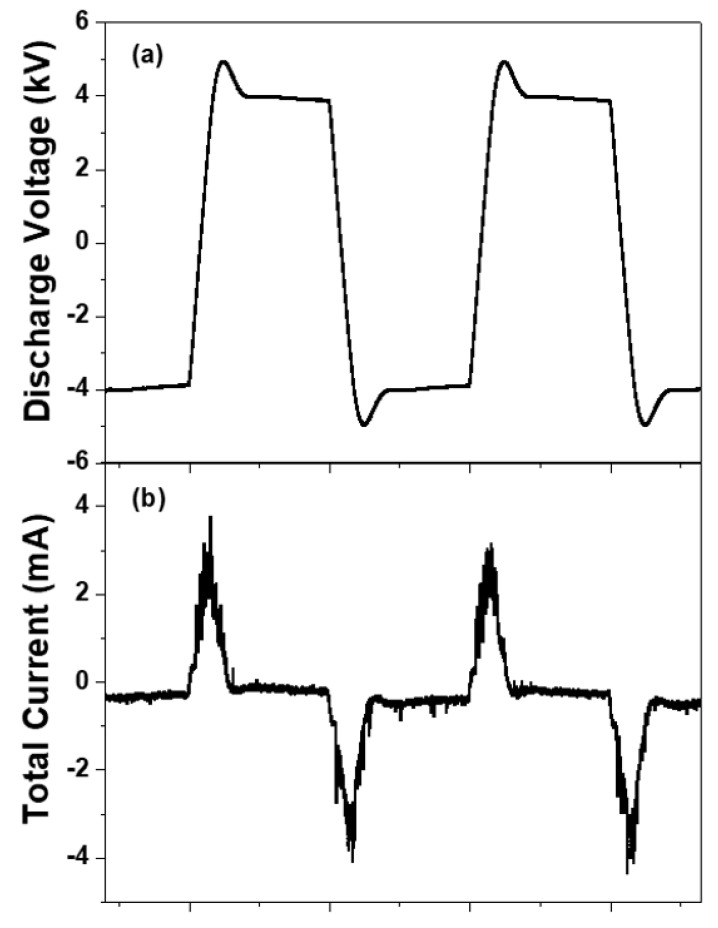
Waveforms of (**a**) discharge voltage and (**b**) total current (= discharge current plus displacement current) measured during low temperature DBD between two electrodes.

Figure 3a shows the waveforms of discharge voltage measured during low temperature DBD between the two electrodes. The discharge voltage showed an amplitude of about 8 kV and rising and falling times of around 20 μs.

Figure 3b shows the total current (= discharge current plus displacement current) with reproducibility during the low temperature DBD. It should be noted that the discharge currents are observed only during the period when the polarity of the voltage is changed, meaning that the cylindrical Cu electrode with the quartz tube acted as a capacitor and, as such, the discharge was spontaneously terminated within a few µs after the discharge was ignited. In other words, the wall charges accumulating in the dielectric layer located below the cylindrical Cu electrode, i.e., quartz tube in this experiment, may play a role not only in terminating the current discharge, but also in facilitating the production of the ensuing discharge. Accordingly, unlike the DC discharge currents, the DBD currents flowing only during a very short period within the one voltage pulse would enable a production of low temperature discharge. Electrical power consumptions of DBD and DC structures are measured using digital power meter. As a result, the consumption powers are 260 W for DBD structure and 560 W for the DC structure, respectively, implying that the DBD structure consumes a low electrical power during the SPP process.

**Figure 4:**
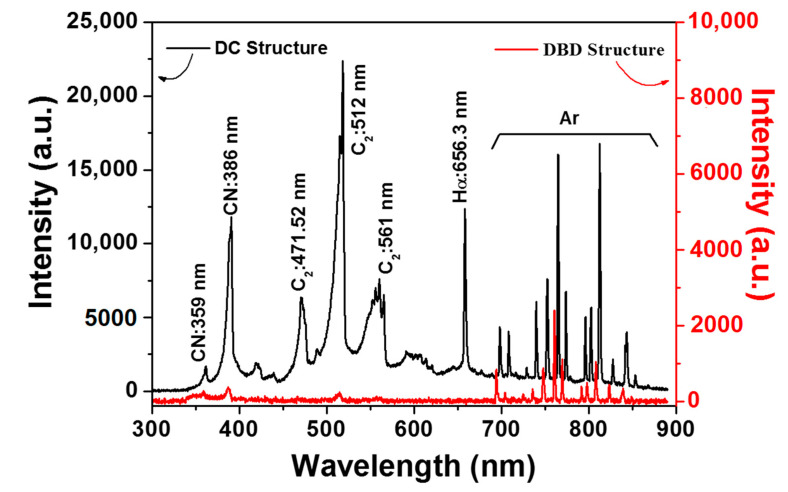
Optical emission spectra (OES) measured during plasma polymerization in the DBD structure compared to the previous DC structure.

Figure 4 shows the optical emission spectra (OES) measured during plasma polymerization in the proposed DBD structure compared to the previous DC structure. It notes that the intensity of the DBD structure is much weaker than that of the DC structure due to its weak emission. In general, multiple excited Ar lines ranging from 698 to 854 nm are observed due to the Ar plasma [30]. Molecular emission peaks of CN and C_2_ are observed, which are related to excitation or dissociation of liquid aniline monomer induced due to the impact of electrons. The main CN peak is 386 nm, while the CN violet system is 359 nm [31]. The CN emission spectra refer to the connection of the benzene ring with an amine group. The emission spectra of C_2_ are 471.52, 512 and 561 nm, which are typically involved in swan bands observed in the combustion of hydrocarbons in organic materials [32,33]. It also notes that the emission peak of C_2_ is related to the combination of C or the degradation of CH and CN. Thus, an increase in the emission peak of C_2_ implies an increase in the probability of synthesis of carbon nanoparticles from liquid aniline monomers, that is, carbonization. Consequently, as shown in Figure 4, the emission peaks of C_2_ in the DBD structure are observed to be significantly reduced compared to those of C_2_ in the DC structure, indicating that carbonization of liquid aniline monomers could be significantly reduced due to low temperature DBD. Furthermore, in the DBD structure, there is no hydrogen Balmer line of H corresponding to 656.3 nm indicating dissociation of liquid aniline monomers [31]. These results confirm that the proposed DBD structure can significantly suppress carbonization.

**Figure 5:**
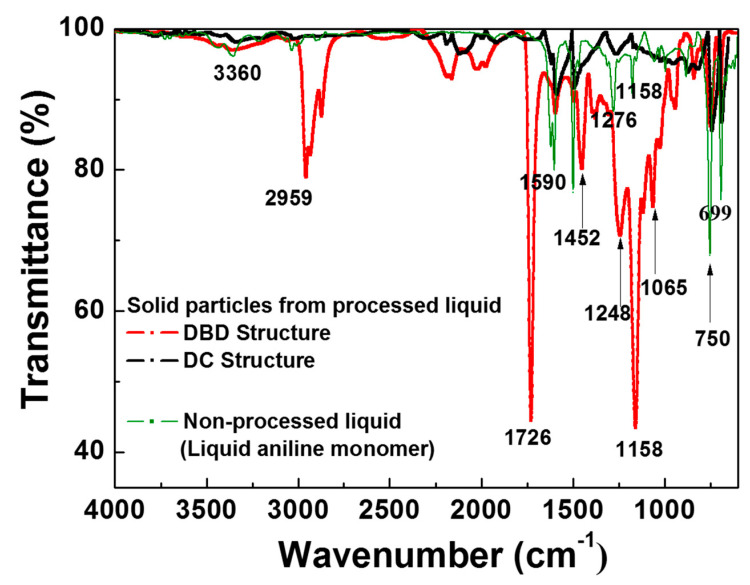
Comparison of FTIR spectra of PANI synthesized in two different discharge electrode structures such as the proposed DBD and previous DC structures, including liquid aniline monomer.

In the FTIR spectra of non-processed liquid aniline monomer in Figure 5, peaks of 1493 and 1276 cm^−1^ are assigned to the C=C and C–N bonds existing in the non-processed liquid, i.e., liquid aniline monomer. Figure 5 also shows the comparison of FTIR spectra of PANI synthesized in two different electrode structures such as the proposed DBD and previous DC structures. The detailed FTIR peaks of the PANI in Figure 5 are listed in Table 1. The PANI synthesized in the proposed DBD structure was observed to have peaks at 699 cm^−1^ and 750 cm^−1^ of C–H out of plane bending mode. The peaks of 1248 cm^−1^ and 1452 cm^−1^ are assigned to the primary aromatic amine C–N bending and C=C aromatic ring stretch mode, respectively. The peak at 2959 cm^−1^ is assigned to the C–H asymmetric stretching mode, whereas the peak at 3360 cm^−1^ is the N–H stretching mode. The peaks of C–O indicate the bond of alcohol for washing PANI for measurement. The C=C and C–N bonds corresponding to the peaks of 1493 and 1276 cm^−1^ in the liquid aniline monomer are considerably reduced in the PANI synthesized by the previous DC structure, but are increased in the PANI synthesized by the proposed DBD structure [34].

Most of measured peaks of the PANI synthesized by the proposed DBD structure are more intense than those of the PANI synthesized by the previous DC structure, meaning that an amount of chemical bonds relative to PANI increase due to the low temperature discharge growth condition under the DBD structure. In the N–H peak intensities at 3360 cm^−1^, both electrode conditions show a slight difference. For the DBD case, however, the peak of 1248 cm^−1^ is prominent, implying that the PANI has lots of C–N bonds, which are related to an increase in the bonds of the benzenoid and quinoid rings.

**Figure 6:**
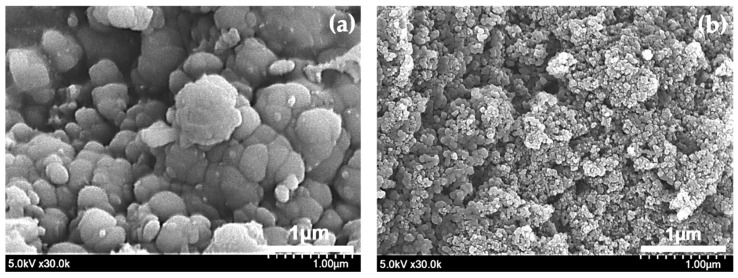
FE-SEM images of PANI nanoparticles synthesized in two different discharge electrode structures; (**a**) proposed DBD structure and (**b**) previous DC structure.

Figure 6a,b shows the FE-SEM images of the PANI nanoparticles synthesized for 3 h in the proposed DBD and previous DC structures, respectively [29]. As can be seen in Figure 6a, the sizes of the PANI nanoparticles are observed to increase significantly compared to those of the DC structure shown in Figure 6b. In addition, it is confirmed that the nanoparticles having a wide variety of sizes are synthesized in the proposed DBD structure. In the previous DC structure, as shown in Figure 6b, the synthesized PANI nanoparticles show a tiny spherical-type nano size structure. Average sizes of PANI nanoparticles for the DBD and DC structures are 277 nm and 28 nm. It can be inferred that fragmentation of the aniline monomer occurs severely due to the strong DC discharge, resulting in the production of many carbonization reactants as well as PANI nanoparticles. It should be addressed that the PANI nanoparticles were effectively synthesized under the low temperature DBD. In addition, it means that the characteristics of the PANI nanoparticles can be controlled according to the discharge characteristics.

**Figure 7:**
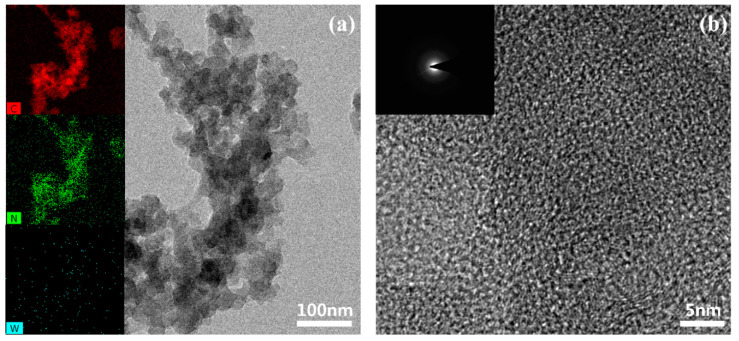
HR-TEM images, SAED pattern and EDS of synthesized PANI by the proposed DBD structure (**a**,**b**) with different magnifications of HR-TEM images; the inset of (**a**) is carbon, nitrogen and tungsten elements measured by EDS, and the inset of (**b**) is the SAED pattern.

Figure 7a,b shows the HR-TEM image and magnified HR-TEM image of PANI synthesized by the proposed DBD structure. EDS analysis in the inset of Figure 7a shows that synthesized PANI includes carbon and nitrogen elements but little tungsten element. It indicates that the erosion of the W electrode is significantly reduced due to the low temperature discharge under the DBD structure. As can be seen in Figure 7b, the specific ordered structure in PANI is not discovered and, as such, the SAED pattern indicates an amorphous pattern. The proposed DBD structure shows the possibility of synthesizing polymer nanoparticles effectively due to the low temperature DBD.

**Figure 8:**
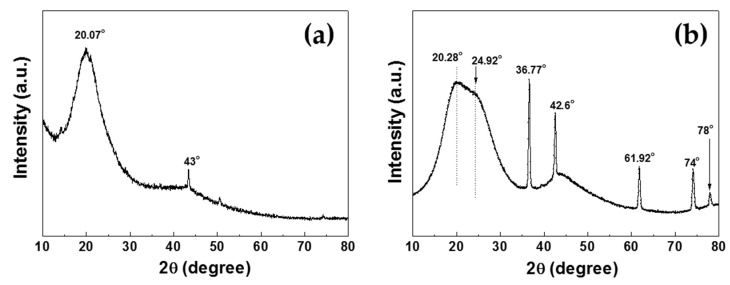
XRD patterns of PANI synthesized in two different discharge electrode structures, such as (**a**) proposed DBD and (**b**) previous DC structures.

Figure 8a,b shows the XRD patterns of PANI synthesized in two different discharge electrode structures such as the proposed DBD and previous DC structures, respectively. In the proposed DBD structure of Figure 8a, the intensive XRD peak was detected at 20.07°, corresponding to the (020) crystalligrphic plane, whereas in the previous DC structure, intensive XRD peaks were detected at 20.28° and 24.92°, corresponding to the (020) and (200) crystalligraphic planes, respectively [35]. Both peaks corresponding to PANI NPs are broad, meaning that these structures are amorphous, but the shape of the XRD pattern in the proposed DBD structure is narrower than in the previous DC structure. In addition, in order to investigate the degree of crystalline for the PANI NPs synthesized by the DBD and DC strucutres, we calculated the values of the full width at half maximum (FWHM) and crystalline size using the Scherrer equation [36] based on the XRD data of Figure 8. The resultant values of both FWHM and crystalline size are given in Table 2. In Table 2, the values of FWHM for (202) crystallographic plane reflections were 5.05° for the DBD structure and 7.15° for the DC structure, respectively. This result indicates the increase in crystalline degree for the PANI NPs synthesized in the DBD structure. In addition, the peaks of tungsten carbide (WC) were observed only at 43°, corresponding to the (200) crystallographic plane’s refections of WC in the proposed DBD structure, whereas various peaks of tungsten carbide were observed at 36.77°, 42.6°, 61.92°, 74° and 78°, corresponding to the (111), (200), (220), (311) and (222) crystallographic planes of WC in the previous DC structure [5,37,38]. These results confirm that the erosion of the tungsten electrode is considerably reduced due to the low temperature discharge under the proposed DBD structure.

## 4. Conclusions

This paper investigates the effects of the dielectric barrier discharge (DBD) on the characteristics of PANI NPs synthesized by a SPP with an Ar gas bubble channel. By adopting a new electrode structure featuring the cylindrical Cu electrode of an external quartz tube with a bubble block plate, a low temperature DBD is produced through an Ar gas bubble channel in liquid aniline monomers for synthesizing PANI NPs. As a result, PANI NPs are successfully synthesized under the low-temperature DBD in liquid aniline monomers. The evolution of the DBD is closely related to the upward movement of the bubble channel, and the active interaction between the Ar channel plasma and the liquid aniline monomer for plasma polymerization would occur mainly in the ascended discharge zone, which is monitored by the ICCD. As shown by the FTIR, PANI NPs synthesized by DBD have lots of C–N bonds, implying the increased bonds of benzenoid and quinoid rings and reduced destruction of liquid aniline monomers. Accordingly, it is observed that the carbon contents, as well as the erosion of the W electrode, can be significantly reduced due to the low temperature DBD. Furthermore, FE-SEM reveals that the average size of PANI NPs grown in the DBD structure is significantly larger than in the previous DC structure. In conclusion, it has been demonstrated that, by applying the proposed DBD electrode structure to the synthesis of PANI NPs using a solution plasma process with an Ar gas bubble channel, the carbonization of aniline monomers and the erosion of electrodes can be significantly reduced. It is expected that the adoption of the proposed DBD electrode structure in a solution plasma process with a gas bubble channel can contribute to improving the quality of the synthesized nanoparticles, especially organic nanoparticles.

## Figures and Tables

**Table 1:** FTIR peaks of PANI synthesized in both DBD and DC structures.

Wavenumber	Vibration Mode
699 cm^−1^	C–H out of plane bending
750 cm^−1^	C–H out of plane bending
1065 cm^−1^	C–O stretching
1158 cm^−1^	C–O stretching
1248 cm^−1^	C–N bending
1452 cm^−1^	C=C ring stretching
1726 cm^−1^	C–O stretching
2959 cm^−1^	C–H asymmetric stretching
3360 cm^−1^	N–H stretching

**Table 2:** Full width at half maximum (FWHM), crystalline size of PANI nanoparticle powders synthesized in two different discharge structures, DBD and DC structures.

Electrode Strucure	Lattice Diffraction	2θ (Degree)	FWHM (Degree)	Crystalline Size (nm)
DBD structure	020	20.07°	5.05°	1.58
DC structure	020	20.08°	7.15°	1.11
	200	24.92°	8.15°	0.98
